# Factors contributing to the temperature beneath plaster or fiberglass cast material

**DOI:** 10.1186/1749-799X-3-10

**Published:** 2008-02-25

**Authors:** Michael J Hutchinson, Mark R Hutchinson

**Affiliations:** 1 Department of Orthopaedics, University of Illinois at Chicago, Chicago, Illinois, USA

## Abstract

**Background:**

Most cast materials mature and harden via an exothermic reaction. Although rare, thermal injuries secondary to casting can occur. The purpose of this study was to evaluate factors that contribute to the elevated temperature beneath a cast and, more specifically, evaluate the differences of modern casting materials including fiberglass and prefabricated splints.

**Methods:**

The temperature beneath various types (plaster, fiberglass, and fiberglass splints), brands, and thickness of cast material were measured after they were applied over thermometer which was on the surface of a single diameter and thickness PVC tube. A single layer of cotton stockinette with variable layers and types of cast padding were placed prior to application of the cast. Serial temperature measurements were made as the cast matured and reached peak temperature. Time to peak, duration of peak, and peak temperature were noted. Additional tests included varying the dip water temperature and assessing external insulating factors. Ambient temperature, ambient humidity and dip water freshness were controlled.

**Results:**

Outcomes revealed that material type, cast thickness, and dip water temperature played key roles regarding the temperature beneath the cast. Faster setting plasters achieved peak temperature quicker and at a higher level than slower setting plasters. Thicker fiberglass and plaster casts led to greater peak temperature levels. Likewise increasing dip-water temperature led to elevated temperatures. The thickness and type of cast padding had less of an effect for all materials. With a definition of thermal injury risk of skin injury being greater than 49 degrees Celsius, we found that thick casts of extra fast setting plaster consistently approached dangerous levels (greater than 49 degrees for an extended period). Indeed a cast of extra-fast setting plaster, 20 layers thick, placed on a pillow during maturation maintained temperatures over 50 degrees of Celsius for over 20 minutes.

**Conclusion:**

Clinicians should be cautious when applying thick casts with warm dip water. Fast setting plasters have increased risk of thermal injury while brand does not appear to play a significant role. Prefabricated fiberglass splints appear to be safer than circumferential casts. The greatest risk of thermal injury occurs when thick casts are allowed to mature while resting on pillow.

## Background

The first recorded use of plaster in a medical situation was in the 9^th ^century A.D. in the Arabic world [1]. More modern use is credited to Antonius Mathyson, a Dutch medical officer, who initiated the use of plaster impregnated bandages for the treatment of musculoskeletal injuries in 1852 [1]. Currently, plaster and fiberglass casts are commonly used to immobilize fractures, correct deformities, splint limbs, and to immobilize the spine [2].

**Figure 1:**
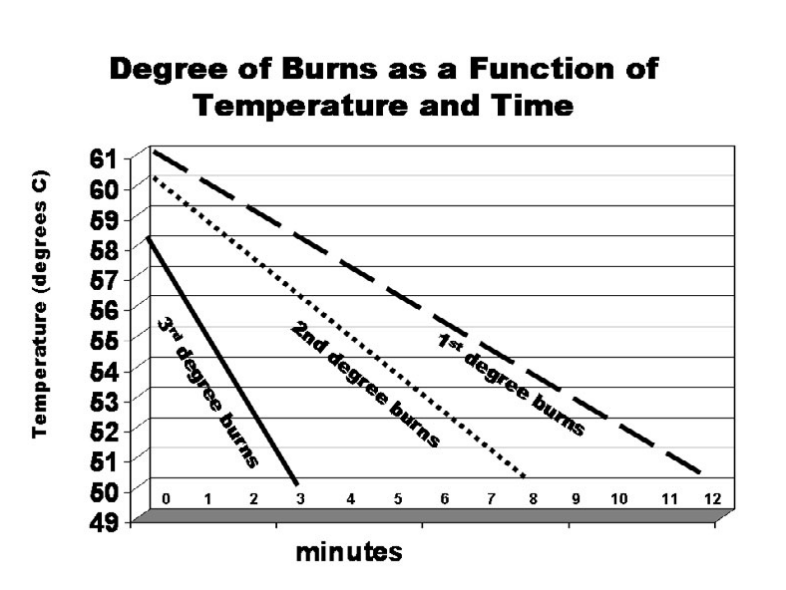
**Based on data from Williamson C, Scholtz JR.** (1949) Time-Temperature relationships in thermal blister formation. *J Invest. Dermatol*. 12: 41–47; this figure represents the time-temperature relationship to create burns on skin.

When cast materials harden, an exothermic reaction occurs causing the temperature within and beneath the cast material to rise. In some cases the temperature rises to dangerous levels that can risk thermal injury [3] (Figure 1). Standard teaching regarding safe casting includes recommendations such as using luke-warm water with plaster casts, cool water with fiberglass casts, and padding appropriately to avoid sharp edges or cast pressure points. Relatively few studies are available that evaluate the effect of various factors as they relate to the temperature beneath fiberglass and plaster casts [1,4-6]. The purpose of this study was to evaluate a number of variables including brand, type of material, thickness, dip water temperature using modern plaster and fiberglass materials relative to their impact on the temperature beneath the cast. Our a-priori hypothesis was that increased layers of both plaster and fiberglass would increase the temperature while increased layers of cast padding would be protective. In addition it was felt that elevated dip water temperature would increase the ultimate temperature beneath the setting cast material. We did not expect to see significant differences between slow and fast setting plasters, and only mild but not dangerous differences between plaster and fiberglass.

## Methods

Three types of plaster (Johnson & Johnson Specialist Fast Plaster 4 inch rolls, Johnson & Johnson Specialist Extra-fast Plaster 4 inch rolls, and Johnson & Johnson Specialist Fast Plaster 4 inch splints; *Johnson & Johnson, New Brunswick, New Jersey*) and two brands of fiberglass (3 M Scotchcast Fiberglass 4 inch rolls: 3 M Inc, *3 M Center, St. Paul, Minnesota*; and Delta-Lite Fiberglass 4 inch rolls: *Johnson & Johnson, New Brunswick, New Jersey*) were used to evaluate the effect of varying the number of cast layers. Prefabricated fiberglass splints which included their own foam padding were also studied. (*3 M Center, St. Paul, Minnesota*) Each was applied over the same diameter polyvinyl chloride (PVC) tube with a thermometer bulb lying on its surface, above the PVC tube, but beneath the cast and padding. Our routine construct had the PVC tube fixed to a table with the casted end lying beyond with air all around. When assessing the maturation on a pillow, the construct was removed and laid on a pillow with the thermometer lying nearest the pillow. The thermometer was selected due to its sensitivity in the temperature range being evaluated. The temperature of the PVC tube was allowed to equilibrate to 32 degrees C prior to the application of any material. In each case a single layer of cotton stockinette was applied followed by predetermined amounts of cast padding and cast material. Reproducibility of measures was assessed by repeating the same construct on three separate occasions and comparing the exact temperature measurements at fixed time intervals.

After confirming reproducibility of measures, the experimental variables included; comparing the effect of variable casting materials, various thickness (number of layers) of casting material (7–12 for fiberglass, and 12–20 for plaster), various types and thicknesses of cast padding (Cotton Webril 1–5 layers; Procel Bubblewrap 1–3 layers), two different dip water temperatures (32 and 37 degrees Celsius), and the effect of allowing the cast to mature while lying on a pillow. Room temperature and humidity were maintained with a restricted range (25–27 degrees Celsius, 32–33% ambient humidity). The dip time was consistent in allowing the material to be saturated and allow all bubbles to be expressed. Cast molding was maintained consistent for all applications by allowing no more than ten seconds of rubbing and molding after final application of material. Selection of specific ranges regarding water temperature, cast thickness, and amount of padding was based on usual clinical practice. The dip water was routinely changed to assure non-contamination with previous plaster material. Temperature readings beneath the cast material were assessed at 1 min, 5 minutes, 10 minutes, 15 minutes, 20 minutes, and if needed at 25 and 30 minutes until peak temperature occurred. Two separate observers confirmed the temperature readings.

## Results

**Figure 2:**
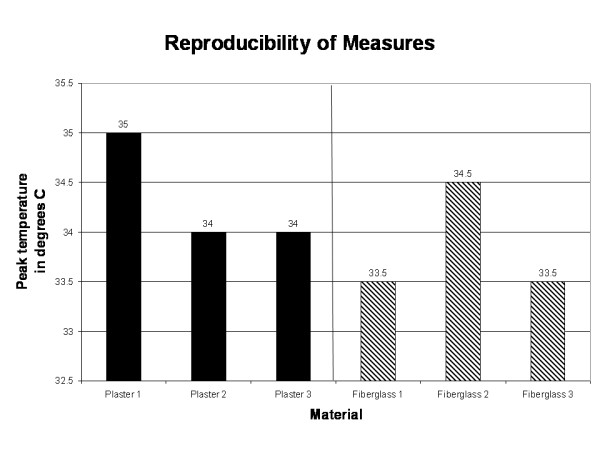
Reproducibility of measures testing was performed for both plaster and fiberglass and revealed test-retest consistency to within one degree.

Outcomes data are documented in Table 1. Tests 1–3 represent the reproducibility of measurements test using 12 layers of plaster, a single layer of Webril padding, and a dip water temperature of 32 degrees Celsius (Figure 2). Outcomes of reproducibility of measures test reveal consistency of measurements within 1 degree for plaster cast material at all times measured; therefore, all variations beyond 1 degree in other measurement tests were deemed to be significant. Tests 10–12 represent the reproducibility of measurements test using 7 layers of fiberglass cast material, a single layer of Webril padding, and a dip water temperature of 32 degrees Celsius. Outcomes of reproducibility measures reveal a consistency within 1 degree for fiberglass cast material for all times measured (Figure 2).

**Table 1:** Data table including variables and outcomes

							***Temperature Beneath Cast (at time in (m) minutes)***									
							
Test #	Cast Material	Layers of cast	Cotton Stockin-ette Present?	Cast Padding Type	Layers of Cast pad	H_2_0 Dip Temp (°C)	1 m	5 m	10 m	15 m	20 m	25 m	30 m	35 m	40 m	Peak
**Repeated Tests for Plaster and Fiberglass**																
1	SFPR	12	Yes	Webril	1	32	27	27.5	28.5	34	35	34	X	X	X	35
2	SFPR	12	Yes	Webril	1	32	28	28	28.5	31	34	34	X	X	X	34.5
3	SFPR	12	Yes	Webril	1	32	29	29	29	32	34	33.5	X	X	X	34
10	DLFR	7	Yes	Webril	1	32	31.5	33	33.5	31	X	X	X	X	X	33.5
11	DLFR	7	Yes	Webril	1	32	31.5	33	34.5	31.5	X	X	X	X	X	34.5
12	DLFR	7	Yes	Webril	1	32	30	31.5	32	30	X	X	X	X	X	33.5
**Effect of Layers of Cast Material on Temperature Underneath**																
4	SFPS	16	Yes	Webril	1	32	27.5	29	28.5	29	32	33.5	X	X	X	33.5
5	SFPS	12	Yes	Webril	1	32	29.5	29.5	29	29.5	31	33.5	X	X	X	33.5
6	SFPS	20	Yes	Webril	1	32	30	30	30	30.5	32.5	33.5	X	X	X	35.5
7	SEFPR	12	Yes	Webril	1	32	28	30	35	41	38	X	X	X	X	41
8	SEFPR	16	Yes	Webril	1	32	30	32.5	40.5	41	36.5	X	X	X	X	41
9	SEFPR	20	Yes	Webril	1	32	33	37	46.5	45	40	X	X	X	X	46.5
14	DLFR	10	Yes	Webril	1	32	32	33.5	36	32.5	X	X	X	X	X	36
15	DLFR	10	Yes	Webril	1	32	32.5	35	38	36	X	X	X	X	X	38
16	DLFR	12	Yes	Webril	1	32	31	33	39	38	X	X	X	X	X	39
**Effect of Cast Padding Thickness and Types**																
21	DLFR	7	Yes	Webril	3	32	31.5	34	34.5	33	X	X	X	X	X	34.5
22	DLFR	7	Yes	Webril	5	32	31.5	34	33.5	32	X	X	X	X	X	34
23	3MSFR	7	No	Procel	1	32	30	34.5	37	33	X	X	X	X	X	37
24	3MSFR	7	No	Procel	3	32	28	30	34	32	X	X	X	X	X	34
25	SFPR	20	Yes	Webril	1	39	29.5	30	32.5	37.5	38	35	X	X	X	38
26	SFPR	20	Yes	Webril	3	39	31.5	32	35.5	42.5	44	41	X	X	X	44
27	SFPR	20	Yes	Webril	5	39	33	33	36	42.5	44	41	X	X	X	44
**Pillow Effect**																
28	SFPR	20	Yes	Webril	1	39	32	32.5	36.5	46.5	53	54	53	50.5	48	54
29	SFPR	12	Yes	Webril	1	32	29	29	31.5	32.5	43	45.5	45.5	X	X	45.5
**Effect of Dip Water, Temp, Splints, etc**.																
13	3MSFR	7	Yes	Webril	1	32	32	36	36.5	32	X	X	X	X	X	36.5
17	DLFR	7	Yes	Webril	1	39	34	36.5	36.5	33	X	X	X	X	X	36.5
18	3MSFR	7	Yes	Webril	1	39	35	39.5	37.5	33	X	X	X	X	X	39.5
19	3MSFR	7	No	Prepadded	1	39	34	32	31.5	30	X	X	X	X	X	34
20	3MSFR	7	No	Prepadded	1	32	27	30	35	33	X	X	X	X	X	35
30	SFPR	20	Yes	Webril	1	39	33	34	39	43	42	39	X	X	X	43
31	SFPR	12	Yes	Webril	1	39	29	30	31	34	36	35.5	X	X	X	36

Presents the data from all tests performed. The purpose of the test is described in the text and grouped by numbers (i.e., tests 1–3 and 10–12 are reproducibility of measures tests for plaster and fiberglass respectively. Data organized in columns represents controlled and variable factors including: plaster type, material thickness, padding thickness, dip water temperature, dip water purity, ambient humidity and temperature, as well as the outcomes measure of temperature over time.

Tests 4–6 and 7–9 document the effect of increasing thickness of plaster casts with Johnson & Johnson Specialist Fast Plaster rolls and Johnson & Johnson Extra-fast Plaster rolls. Outcomes reveal minimal temperature effect of increasing plaster thickness with the slower setting Johnson & Johnson Fast Plaster; however, when the fast setting Johnson & Johnson Extra-fast Plaster is used, there is a significant elevation of 5 degrees when 20 layers is applied compared to 12 or 16 layers. Clearly the type of cast material (fast or extra-fast setting) is another important factor. In each case the extra-fast setting plaster revealed increased temperatures from 8–11 degrees Celsius respectively. Indeed the Johnson & Johnson Extra-fast plaster with 20 layers of thickness approached potentially dangerous levels with a maximum temperature of 46.5 degrees Celsius at ten minutes for a limited time period. Peak temperatures for extra-fast plaster occurred between 10–15 minutes earlier than with fast setting plaster.

Test 13 was performed to compare to Tests 10–12 and evaluate the effect of different fiberglass brands. The fiberglass from 3 M appears to mature at about the same pace as Johnson and Johnson Delta Lite but reaches a peak temperature of about 2 degrees greater. This did not approach dangerous levels relative to thermal injury (over 49 degrees for an extended period).

Tests 10–12 and 14–16 were performed to evaluate the effect of increased thickness of fiberglass casts. Beneath the fiberglass cast of 7, 10, and 12 layers, outcomes revealed a progressive increase in temperature of about 2 degrees each up to 39 degrees Celsius. This did not approach dangerous levels.

Tests 17, 18, and 25 are compared to Tests 10–13 and Test 6 to evaluate the effect of increasing dip water temperature from 32 to 39 degrees Celsius. Outcomes reveal that increasing dip water temperature increases the ultimate peak temperature beneath the cast by 2–3 degrees for all three types of cast material tested (Johnson and Johnson Fast-Plaster, Johnson and Johnson Delta Lite fiberglass, and 3 M fiberglass). The highest peak temperature of 39.5 degrees was achieved by the 7 layers of 3 M fiberglass dipped into 39 degree dip water. This did not approach dangerous levels.

Tests 13, 18–20 were compared to evaluate the effect of prefabricated 3 M fiberglass splints relative to similar thickness 3 M fiberglass casts at temperatures of 32 and 39 degrees. Results revealed a slight decrease in peak temperature of 1.5 to 5 degrees when comparing the prefabricated fiberglass splints compared to rolled casts.

Tests 10–12, 21–22 were compared to evaluate the effect of varying padding thickness beneath a fiberglass cast. Measurements were made with 1, 3 and 5 layers of cotton Webril. Results revealed no effect of cotton Webril padding thickness beneath the fiberglass material. Peak temperatures with a single layer of padding averaged 34 degrees compared to the measured peak temperature with three layers of padding of 34.5 degrees and with five layers of 34 degrees.

Tests 25–27 were performed to evaluate the effect of varying padding thickness beneath plaster. Temperature measurements were made using one, three, and five layers of cotton Webril beneath the slower setting plaster. Results revealed increased temperatures by 6 degrees with either the three or five layer increased padding thickness when compared to the single layer of cotton padding. The peak temperature of 44 degrees would be dangerous if maintained for an extended period of time. In this experiment peak temperature was present for less than five minutes.

Tests 23 and 24 compared the effect of increasing thickness (1–3 layers) of the non-Webril, Procel, "bubblewrap" padding. Results revealed a three degree decrease in temperature with the thicker wrapping. In general, temperatures achieved beneath the Procel padding were similar to those achieved with cotton Webril padding.

**Figure 3:**
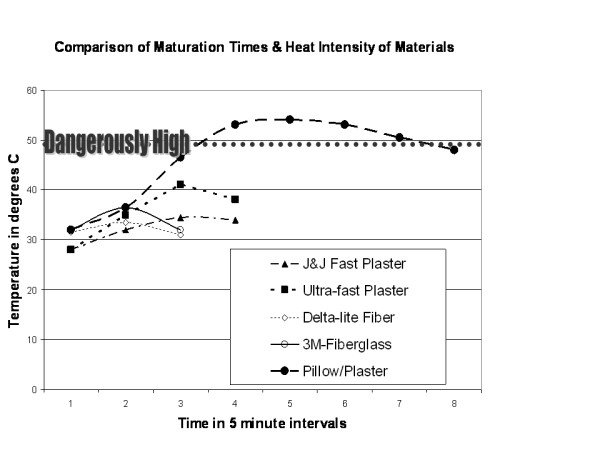
The most significant finding of the study revealed that thick fast setting plaster allowed to mature on a pillow increased temperatures beneath the cast to dangerous levels which would place a patient at risk of severe burns.

Test 28 and 29 were performed to evaluate the effect of laying a maturing plaster cast on a pillow. In test 28, our standard protocol was maintained with a single layer of Webril padding beneath 20 layers of the slow setting plaster dipped into the warmer 39 degree dip water. This was done to maximize the effect. An even worst case scenario could be imagined if extra-fast setting plaster was used. In test 29, our standard protocol was maintained with a single layer of Webril beneath 12 layers of the slow setting plaster immersed in 32 degree dip water. In both tests the temperature was elevated for an extended period of time; indeed, twenty layers of slow setting plaster dipped in warm water exceeded the pre-determined dangerous level of Williamson (over 50 degrees) for an extended period of time (over 25 minutes) (Figure 3).

## Discussion

A number of complications from casting, padding, and the use of plaster bandages have been described including deformity, skin injuries, rashes, compartment syndrome, and burns [7]. The mechanism of these injuries include: improperly and irregularly applied padding that leads to pressure sores beneath the cast, inadequate padding material at the ends of the cast leading to sharp edges and skin irritation, aggressive cast molding that leads to pressure sores beneath the cast, inadequate casting material leading to cast breakdown and loss of control of the unstable fracture, tight application of casting material or failure to allow for underlying injury swelling leading to compartment syndrome, and hot dip water leading to elevated setting temperatures and skin burns [2,8].

The purpose of this study was to evaluate various factors and their effect on ultimate temperature beneath various casting materials and techniques. In a study of thermal injuries to the skin, Williamson [3] assessed the effect of elevated temperatures on the skin and the risk of 1^st^, 2^nd^, and 3^rd ^degree burns relative to time exposure (Figure 1). While this study did not use casting as their model, their study showed that maintaining temperatures of over 49 degrees for an extended period of time risked 1^st ^degree burns if the exposure was longer than 2–3 minutes, 2^nd ^degree burns if the exposure was longer than 8 minutes, and 3^rd ^degree burns if the exposure was longer than 12 minutes. This study was our basis of defining temperatures beneath a cast of greater than 49 degrees Celsius for an extended period of time as dangerous.

A number of authors have noted the importance of monitoring dip water temperature and its effect on level of the temperature achieved by the exothermic reaction [1,5,9,10]. Indeed, Lavallette et al. [5,6] demonstrated a direct effect with dip water temperature, the length of time the plaster is kept in the dip water, and the risk of burns. Our studies confirm the findings of Lavallette that dip water temperature can play a key role in the ultimate temperature beneath the cast. Kaplan [1] showed that temperature elevations could be related to the plaster being dipped too briefly and the water being squeezed too aggressively out of the plaster. The water itself helps to release the heat, and if there is not enough, the plaster gets hotter. In this study, we attempted to control this factor by maintaining a strict regimen of time in dip water, allowing bubbles to exude, and gentle squeezing the water out prior to application. In addition, in this study we attempted to maintain uniformity by molding the material for a defined amount for in each test sample. Regarding fiberglass cast material, Selesnick and Griffiths [10] recommended using only cool dip water to reduce the chance of burns. In this study, we used both the 32 and 39 degree temperature dip water for plaster and fiberglass to allow direct comparisons of the materials. Regarding the effect of dip water temperature, this study confirms a direct relationship with increasing dip water temperature from 32 to 39 degrees Celsius and the ultimate peak temperature beneath both plaster and fiberglass casts. The comparison of plaster material revealed an increased in peak temperature of 2 degrees and the comparison of 3 M fiberglass material revealed an increase of 3 degrees related to the higher dip water temperature. It is possible that even greater dip water temperatures could increase the ultimate temperature beneath the cast. Admittedly, this is hypothesis that was not confirmed within our range of constructs.

Dirty dip water and ambient humidity have also been implicated as contributing to temperatures beneath maturing casts. Lavalette [5,6] and Ganaway [4] proposed that plaster residue in the dip water might also play a role in elevating cast temperature and broadening the time-temperature curve; i.e., maintaining the peak temperature for a longer period. In our study this factor was controlled by maintaining fresh dip water for each test. In the orthopaedist's office or emergency room that is doing a lot of casting, this factor may need to be accounted for to minimize the time that the temperatures beneath a cast are elevated. Ganaway [4] felt that ambient humidity also played a role in the ultimate cast temperature; therefore, in this study ambient humidity was controlled to within 1%.

Additional factors play significant roles on the ultimate temperature beneath a cast and were controlled variables in this study. They include fast versus slow setting plasters, cast thickness, different brands of material, and the thickness and type of cast padding. Ganaway [4] felt that cast padding played little role in effecting the temperature beneath a cast. Our initial hypothesis was that thicker padding would be protective of the underlying temperature. In contrast what we found was that while increased cast padding had little effect on the fiberglass casts, it had a significant effect of elevated temperatures when additional layers of Webril were applied beneath 20 layers of extra-fast setting plaster. This was exactly opposite of what we had hypothesized. This effect may be explained by increased insulation trapping the heat beneath. The Procel bubblewrap offered little variation compared to Webril when placed beneath a fiberglass cast. Cast padding likely plays a greater role to protect the skin against pressure points than its effect on temperature.

The assessment of temperature beneath prefabricated splints along with its comparison to other forms of casting has not been previously reported. We found that the prefabricated fiberglass splints correlated with reduced temperatures beneath the splint material. This was likely secondary to the absence of circumferential splint material that would trap the heat beneath the material which, in turn, allowed the heat to defervesce laterally and more quickly. This finding would clearly support the premise that these prefabricated splints are safer, relative to thermal injury, than circumferential casting techniques.

Regarding the effect of various plaster materials, our findings agree with those of Ganaway and Hunter [4] which revealed that faster setting plasters have earlier and higher peak temperatures. Comparing different brands of fiberglass (Tests 17 and 18) revealed differences in peak temperatures but not onset of peak temperatures between brands. Neither was noted to achieve dangerous levels of temperature with dip water temperature of 39 degrees Celsius.

Ultimate cast temperature is related to the amount of plaster, its surface area, and the external environment's ability to let plaster lose heat [11]. In this study, we maintained the surface area constant with a standard diameter PVC tube. We then evaluated the effect of varying thickness of cast materials, padding, and external applied material (a pillow). Both Lavalette and Ganaway [4-6] felt that the thickness of plaster played a significant role in peak temperature. Both also agreed that poor cast ventilation (such as an externally applied pillow), would lead to increased peak cast temperature. In our study we found that for all cast materials, plaster or fiberglass, increased thickness led to increased temperatures beneath the cast. However, we found only one construct in which the temperatures achieved and the duration of that intensity fulfilled criteria deemed to be dangerous by Williamson et al. [3]. When the thickest construct of extra-fast plaster (20 layers with 1 layer of Webril) dipped in 39 degree Celsius water was allowed to lie on a pillow through its maturation, temperatures exceeded 50 degrees Celsius for over 20 minutes. Using Williamson's work [3], this would translate to a 3^rd ^degree burn if applied on a human extremity. In Test #9 the temperature beneath the a twenty layer thick extra fast setting plaster dipped in 32 degree water (not on a pillow) peaked at 46.5 degrees Celsius and over 40 degrees for 10 minutes. Indeed when 20 layers of the normal setting cast material was dipped in warmer water (tests 26–27), the peak temperatures achieved 44 degrees and were maintained over 40 degrees for at least 25 minutes. While these did not meet the minimum criteria of exceeding 49 degrees, the thermal exposure over 40 degrees for an extended period of time raises concern.

A potential criticism of this study is our selection of a polyvinyl (PVC) tube model instead of a glass cylinder filled with water as suggested by Lavellette [4]. Previous authors have suggested that internal diffusion of heat by the fluid or by the blood in the human model may serve to defervesce the temperature more quickly and avoid dangerous temperature levels. We don't disagree that this may play a role. Our model allowed the PVC tube to equilibrate to 32 degrees C before each new test and used the hollow, air filled PVC to serve as our diffuser. In addition and unlike Lavellette's study, the size of tubing was selected to mimic the average size of an adult calf or upper arm. This allowed a consistent surface area of casting material. In addition in this study, we did not specifically compare the absolute temperatures achieved by Lavellette or others but rather the effect and trend of altering variables within our model. Our only absolute temperature measurement comparison was performed using the Williamson study [3] regarding what temperatures are necessary to cause thermal injuries to skin. Indeed a number of our constructs raised concern, especially when allowing casts to mature while lying on a pillow. Perhaps a follow-up study placing our thermometer below casts placed in-vivo on volunteers would confirm the absolute temperatures that we report in vitro to be consistent with those seen in vivo.

In summary, a number of studies have evaluated the exothermic reaction that occurs during casting and have looked at the effect of a number of variables on the temperature beneath the cast. Unlike the few studies available on this topic, this study is unique that it included modern materials of fiberglass, prefabricated fiberglass splints, synthetic Procel padding in comparison to the classic plaster and cotton Webril padding. We can conclude the following:

1. Extra fast setting plaster achieves peak temperatures quicker and higher than slower setting plasters.

2. Increased thickness of casting materials (both plaster and fiberglass) are related to increased temperatures beneath the cast.

3. Dip water temperature is directly related to the peak temperature beneath the cast.

4. Brand of fiberglass did not play a significant role in the brands we studied.

5. Prefabricated splints do not achieve the same temperature levels when compared to circumferential casts and, therefore, from a thermal perspective, may be safer.

6. Thickness and type of cast padding did not play a significant role regarding ultimate temperatures beneath the cast in this study. At the thicker levels of padding, it may actually serve as an insulator entrapping additional heat.

7. The greatest risk of thermal injury occurs when a thick cast using warm dip water is allowed to mature while resting on a pillow.
